# Association between objective nutritional indices and malnutrition inflammation score in peritoneal dialysis patients

**DOI:** 10.3389/fnut.2025.1713482

**Published:** 2026-01-08

**Authors:** Xinqi Teng, Xiangyu Yang, Leiyun Wang, Lulu Li

**Affiliations:** Department of Pharmacy, Wuhan No.1 Hospital, Wuhan, Hubei Province, China

**Keywords:** chronic kidney disease, inflammation, malnutrition, malnutrition-inflammation score, objective nutritional indices, peritoneal dialysis

## Abstract

**Objective:**

This study aimed to investigate the associations between objective nutritional indices and the Malnutrition-Inflammation Score (MIS) in patients undergoing peritoneal dialysis (PD)..

**Methods:**

This cross-sectional study enrolled 147 maintenance PD patients. Participants were stratified into low (MIS ≤ 5, *n* = 62) and high (MIS > 5, *n* = 85) malnutrition-inflammation risk groups based on MIS. The objective nutritional indices, such as C-reactive protein-to-albumin ratio (CAR), advanced lung cancer inflammation index (ALI), prognostic nutritional index (PNI), geriatric nutritional risk index (GNRI), and controlling nutritional status (CONUT) score, were calculated. Clinical characteristics, laboratory parameters, and objective indices were compared between the groups. Correlation analyses and multivariable linear regression were used to assess the relationship between MIS and each objective index. The predictive performance of each index for identifying malnutrition risk was evaluated using receiver operating characteristic (ROC) curve analysis.

**Results:**

Patients with an MIS of >5 had significantly lower body mass index (BMI), albumin, lymphocyte percentage, ALI, PNI, and GNRI, as well as significantly higher dialysis vintage, CRP, neutrophil-to-lymphocyte ratio (NLR), CAR, and CONUT scores (all *p* < 0.05). All objective indices showed significant correlations with MIS, with GNRI demonstrating the strongest correlation (*r* = −0.558, *p* < 0.001). These associations remained significant after multivariate adjustment. The ROC analysis indicated that all indices had significant predictive value for an MIS of >5. GNRI showed the highest predictive performance (area under the curve (AUC) = 0.75, 95% CI: 0.67–0.83), with an optimal cutoff value of 92.5 (sensitivity 90.3%, specificity 56.5%), followed by ALI (AUC = 0.72) and PNI (AUC = 0.70).

**Conclusion:**

Objective nutritional indices, particularly GNRI, ALI, and PNI, are significantly associated with MIS and effectively identify PD patients at high risk of malnutrition. These readily available objective tools could serve as practical alternatives to MIS for rapid nutritional assessment and large-scale screening in clinical practice. However, future multicenter, large-scale studies are warranted to validate our findings and determine the optimal index.

## 1 Introduction

Malnutrition and chronic inflammation are prevalent among end-stage kidney disease (ESKD) patients, especially those undergoing dialysis (1, 2). Inflammatory processes can initiate protein-energy wasting (PEW), thereby worsening nutritional decline in patients undergoing dialysis. These two conditions are interrelated and frequently coexist in maintenance dialysis patients, which is referred to as malnutrition–inflammation complex syndrome (MICS) in the literature (2, 3). Previous studies have shown that MICS is strongly associated with increased risks of complications and mortality in dialysis patients (4, 5). Given the significant importance of nutritional status in the prognosis of dialysis patients, early identification of high-risk individuals and timely intervention are essential for improving clinical outcomes (2). Therefore, malnutrition, as defined by different tools, is gaining attention.

The Subjective Global Assessment (SGA) and Malnutrition-Inflammation Score (MIS) are well-established, comprehensive scoring tools used for nutritional assessment in patients undergoing dialysis (3, 6). SGA is based on the subjective evaluation of patients’ gastrointestinal symptoms and physical examination by clinicians. MIS incorporates three objective components based on the SGA, including anthropometric measurements, serum albumin, and total iron binding capacity (TIBC). Studies have shown that the MIS effectively predicts mortality and clinical outcomes in both peritoneal dialysis (PD) and hemodialysis (HD) patients (7, 8). A meta-analysis determined that an MIS of ≥ 5 is optimal for defining PEW, with a significantly higher mortality risk in PD patients (8). A cross-sectional analysis of 2,975 HD patients revealed that an MIS of > 5 was significantly associated with malnutrition, and increased CRP was independently associated with an MIS of > 5 (9).

However, the assessment of MIS partly depends on the operator’s subjective judgment, which limits its application, especially in large-scale implementation (10). Several composite objective indices that combine both nutritional and inflammatory markers—including the C-reactive protein-to-albumin ratio (CAR) (11), advanced lung cancer inflammation index (ALI) (12), prognostic nutritional index (PNI) (13), geriatric nutritional risk index (GNRI) (14), and CONUT score (15)—have been shown to predict mortality and prognosis in chronic kidney disease (CKD) and dialysis patients. Some studies have confirmed that these objective nutritional indices have reliable predictive value for the nutritional status of PD patients (10, 16). Compared to subjective assessment tools, these objective nutritional indices are more convenient and rapid, as they do not rely on the operator’s subjective evaluation. Our study aimed to explore the relationship between MIS and these objective nutritional indices to further confirm its reliability and identify potential surrogate markers for assessing the risk of malnutrition in PD patients.

## 2 Methods

### 2.1 Study population and data collection

Participants were consecutively recruited from patients receiving treatment at the PD Center of Wuhan No.1 Hospital, China, between 1 January 2022 and 31 December 2024. The inclusion criteria were patients aged more than 18 years and on maintenance continuous ambulatory PD for more than 3 months with 3–5 daily exchanges. The exclusion criteria comprised recent peritonitis or other infections within the past month and severe comorbidities, including malignancies, hepatitis, or tuberculosis. Ultimately, 147 patients were included in this study. Data collection encompassed general characteristics, including age, sex, BMI, dialysis vintage, and comorbidities. Nutritional parameters included total protein, albumin, and prealbumin; inflammatory parameters included CRP, white blood cells, neutrophils, and lymphocytes; other laboratory measurements included parathyroid hormone (PTH), TIBC, transferrin saturation, ferritin, transferrin, triglycerides, total cholesterol, low-density lipoprotein cholesterol (LDL-C), hemoglobin, platelets, serum globulin, urea, creatinine, and uric acid.

### 2.2 MIS

We used the MIS to assess the malnutrition risk of PD patients. MIS comprised 10 components, including five components from the medical history (weight change, dietary intake, gastrointestinal symptoms, functional capacity, disease, and their relation to nutritional requirements), three physical examination components (signs of fat and muscle wasting and BMI), and two biochemical parameters (albumin and TIBC). Each component was evaluated using a standardized 4-point severity scale ranging from 0 (normal) to 3 (most severe), with the total MIS calculated by summing all component scores yielding a possible range from 0 to 30 (3). Based on previous studies, an MIS of > 5 was defined as high risk of malnutrition (8, 9). Two dietitians assessed the patients’ MIS following a consistency training session to ensure standardized and reliable scoring.

### 2.3 Assessment of objective nutritional indices

The CAR was calculated as CRP (mg/L) divided by serum albumin (g/dL) (11). The calculation formula for ALI was as follows: ALI = BMI (kg/m^2^) × serum albumin (g/dL) / NLR, where NLR = absolute neutrophil count based on blood samples / absolute lymphocyte count (17). The PNI was calculated using the formula: [10 × serum albumin (g/dL)] + [5 × total lymphocyte count (×10^9^/L)] (13). The calculation formula for GNRI was: GNRI = [14.89 × serum albumin (g/dL)] + [41.7 × (current body weight / ideal body weight (IBW))]. IBW was sex-specific and calculated as: men: height (cm)–100–[(height (cm)–150)/4], women: height (cm)–100–[(height (cm)–150)/2.5]. The ratio of current weight to IBW was capped at 1 if the patient’s weight exceeded their IBW (16). The CONUT score was calculated based on serum albumin, total cholesterol, and total lymphocyte count, as detailed in Supplementary Table S1 (15). All serum albumin values, originally measured in g/L, were converted to g/dL (by dividing by 10) for use in all calculations and result presentations to maintain consistency with the cited formulas.

### 2.4 Sample size calculation

*A priori* sample size calculation was conducted using G*Power 3.1. For a linear multiple regression with an effect size f^2^ = 0.15, *α* = 0.05, power = 95%, and six predictors, the minimum required sample size was 146. Our final sample comprised 147 participants, exceeding this requirement and thus ensuring adequate statistical power for the analysis.

### 2.5 Statistical analyses

Continuous variables were expressed as mean ± SD or median [interquartile range, IQR] based on their distribution. Normality was assessed using the Shapiro–Wilk test. Group comparisons between patients with high (MIS > 5) and low (MIS ≤ 5) malnutrition risk were performed using independent Student’s t-tests for normally distributed variables and the Mann–Whitney U-tests for non-normally distributed variables. Categorical variables were compared using the chi-square tests. Correlation analyses between MIS and other variables were performed using Pearson’s test for normally distributed data and Spearman’s rank correlation for non-normally distributed variables. A multivariable linear regression analysis was performed to assess the independent association between objective nutritional indices and MIS. The normality of the model residuals was assessed via diagnostic plots. Receiver operating characteristic (ROC) curve analysis was performed to assess the discriminatory power of objective indices for malnutrition risk, with optimal cutoff values determined by Youden’s index. All analyses were conducted using IBM SPSS 25.0, with two-tailed *p*-values of <0.05 considered statistically significant.

## 3 Results

### 3.1 Comparison of baseline characteristics

A total of 147 patients participated in the study. According to MIS, 62 patients were classified into the low-malnutrition risk group, while 85 patients were assigned to the high-risk group. Table 1 summarizes the comparative characteristics between the groups. Patients in the high-risk group had significantly lower BMI (21.63 [19.42, 24.60] vs. 23.99 [21.28, 27.12], *p* = 0.004) and albumin levels (3.55 ± 0.47 g/dL vs. 3.84 ± 0.32, *p* < 0.001). They were also older (59.06 ± 12.12 vs. 54.27 ± 11.33, *p* = 0.016) and had a longer dialysis vintage (38.00 [27.00, 71.50] vs. 26.00 [14.00, 35.25], *p* < 0.001). Markers of inflammation were notably higher in the MIS > 5 group, including increased CRP (5.15 [2.34, 9.01] vs. 3.74 [1.88, 5.52] mg/L, *p* = 0.027), higher neutrophil percentage (68.83 ± 8.02% vs. 66.28 ± 6.69%, *p* = 0.043), and elevated NLR (3.61 [2.81, 4.61] vs. 3.30 [2.51, 4.47], *p* = 0.003). Conversely, lymphocyte percentage was significantly lower in the MIS > 5 group (20.25 ± 7.26% vs. 22.94 ± 5.83%, *p* = 0.017). The high-risk group had significantly different values for all composite indices compared to the low-risk group, with higher CAR (1.47 [0.63, 2.34] vs. 0.92 [0.48, 1.39]; *p* = 0.001) and CONUT scores (4.00 [3.00, 5.00] vs. 3.00 [2.00, 4.00]; *p* = 0.009). They also had lower values for ALI (20.97 [14.26, 28.52] vs. 28.48 [17.01, 34.01]), PNI (42.33 [38.22, 46.44] vs. 46.22 [43.28, 49.16]), and GNRI (92.00 [87.00, 97.00] vs. 98.50 [95.00, 101.25]; *p* < 0.001). No significant differences were observed between the groups in sex, cardiovascular disease, prealbumin, white blood cells, hemoglobin, PTH, iron metabolism (except ferritin), lipid profiles, or renal function parameters (urea, creatinine, and uric acid; *p* > 0.05).

**Table 1:** Comparison of characteristics between patients with low malnutrition risk (MIS ≤ 5) and high malnutrition risk (MIS > 5).

Characteristic	MIS ≤5 (*n* = 62)	MIS >5(*n* = 85)	*p*
General characteristics			
Age (years)	54.27 ± 11.33	59.06 ± 12.12	**0.016**
Sex (n, %)			0.091
Male	35 (56.45%)	36 (42.35%)	
Female	27 (43.55%)	49 (57.65%)	
BMI (kg/m^2^)	23.99 [21.28, 27.12]	21.63 [19.42, 24.60]	**0.004**
Dialysis vintage (months)	26.00 [14.00, 35.25]	38.00 [27.00, 71.50]	**<0.001**
Diabetes (n, %)	18 (29.03%)	27 (31.76%)	0.723
Cardiovascular disease (n, %)	47 (75.81%)	61 (71.76%)	0.584
Laboratory measurements			
PTH (pg/mL)	210.00 [86.90, 374.00]	251.00 [97.50, 445.00]	0.630
Serum iron (μmol/L)	19.19 ± 7.21	17.28 ± 8.41	0.155
TIBC (μg/dL)	326.36 ± 52.47	304.40 ± 81.13	0.067
Transferrin saturation (%)	33.13 ± 11.87	32.72 ± 15.38	0.862
Ferritin (μg/L)	71.90 [39.60, 163.00]	111.00 [58.60, 227.00]	**0.035**
Transferrin (g/L)	2.34 [2.00, 2.68]	2.50 [2.20, 2.70]	**0.003**
Triglycerides (mmol/L)	1.70 [1.20, 2.57]	1.42 [0.97, 3.24]	0.475
Total cholesterol (mmol/L)	4.26 ± 1.098	4.33 ± 1.291	0.723
LDL-C (mmol/L)	2.13 ± 0.79	2.26 ± 0.83	0.351
Hemoglobin (g/L)	117.27 ± 17.94	112.31 ± 20.26	0.126
Red blood cells (×10^12^/L)	3.87 ± 0.66	3.71 ± 0.69	0.173
Platelets (×10^9^/L)	199.26 ± 56.05	208.07 ± 72.54	0.426
Serum globulin (g/L)	29.88 ± 3.95	29.78 ± 5.45	0.898
Urea (mmol/L)	17.44 ± 3.94	17.24 ± 5.67	0.813
Creatinine (μmol/L)	975.15 ± 343.71	939.52 ± 302.26	0.507
Uric acid (μmol/L)	387.68 ± 106.06	369.13 ± 97.66	0.275
Nutritional parameters			
Total protein (g/L)	68.24 ± 5.43	64.87 ± 9.91	**0.017**
ALB (g/dL)	3.84 ± 0.32	3.55 ± 0.47	**<0.001**
Prealbumin (mg/L)	379.05 [190.00, 569.05]	310.92 [176.78, 487.70]	0.401
Inflammatory parameters			
CRP (mg/L)	3.74 [1.88, 5.52]	5.15 [2.34, 9.01]	**0.027**
White blood cells (×10^9^/L)	7.01 ± 2.04	6.91 ± 2.23	0.776
Neutrophils (%)	66.28 ± 6.69	68.83 ± 8.02	**0.043**
Lymphocytes (%)	22.94 ± 5.83	20.25 ± 7.26	**0.017**
NLR	3.30 [2.51, 4.47]	3.61 [2.81, 4.61]	**0.003**
Nutritional indices			
CAR	0.92 [0.48, 1.39]	1.47 [0.63, 2.34]	**0.001**
ALI	28.48 [17.01, 34.01]	20.97 [14.26, 28.52]	**<0.001**
PNI	46.22 [43.28, 49.16]	42.33 [38.22, 46.44]	**<0.001**
GNRI	98.50 [95.00, 101.25]	92.00 [87.00, 97.00]	**<0.001**
CONUT score	3.00 [2.00, 4.00]	4.00 [3.00, 5.00]	**0.009**

MIS, malnutrition-inflammation score; BMI, body mass index; PTH, parathyroid hormone; TIBC, total iron-binding capacity; LDL-C, low-density lipoprotein cholesterol; ALB, albumin; CRP, C-reactive protein; NLR, neutrophil-to-lymphocyte ratio; CAR, C-reactive protein-to-albumin ratio; ALI, advanced lung cancer inflammation index; PNI, prognostic nutritional index; GNRI, geriatric nutritional risk index; CONUT score, controlling nutritional status score. *p*-values < 0.05 are indicated in bold.

### 3.2 Correlations between MIS and objective nutritional indices

As shown in Table 2, multiple indicators demonstrated significant correlations with MIS. Among clinical parameters, dialysis vintage (*r* = 0.360, *p* < 0.001), age (*r* = 0.215, *p* = 0.009), and BMI (*r* = −0.302, *p* < 0.001) were significantly correlated with MIS. Nutritional biomarkers, including albumin (*r* = −0.459, *p* < 0.001), total protein (*r* = −0.342, *p* < 0.001), and transferrin (*r* = −0.327, *p* < 0.001), showed significant inverse correlations with MIS. Inflammatory markers CRP (*r* = 0.170, *p* = 0.048) and NLR (*r* = 0.237, *p* = 0.004) exhibited positive correlations, while lymphocytes demonstrated negative correlations (*r* = −0.250, *p* = 0.002). All five composite indices showed significant associations with MIS. ALI (*r* = −0.415, *p* < 0.001), PNI (*r* = −0.466, *p* < 0.001), and GNRI (*r* = −0.558, *p* < 0.001) showed negative correlations, with GNRI exhibiting the strongest correlation. Conversely, CAR (*r* = 0.268, *p* = 0.001) and CONUT score (*r* = 0.328, *p* < 0.001) were positively correlated with MIS.

**Table 2:** Correlation analysis between MIS and specific indicators.

Characteristic	R	*p*
Age	0.215	**0.009**
Sex	0.113	0.173
BMI	−0.302	**<0.001**
Dialysis vintage	0.360	**<0.001**
Total protein	−0.342	**<0.001**
ALB	−0.459	**<0.001**
Ferritin	0.123	0.139
Transferrin	−0.327	**<0.001**
CRP	0.170	**0.048**
Neutrophils	0.152	0.066
Lymphocytes	−0.250	**0.002**
NLR	0.237	**0.004**
CAR	0.268	**0.001**
ALI	−0.415	**<0.001**
PNI	−0.466	**<0.001**
GNRI	−0.558	**<0.001**
CONUT score	0.328	**<0.001**

ALB, albumin; BMI, body mass index; CRP, C-reactive protein; NLR, neutrophil-to-lymphocyte ratio; CAR, C-reactive protein-to-albumin ratio; ALI, advanced lung cancer inflammation index; PNI, prognostic nutritional index; GNRI, geriatric nutritional risk index; CONUT score, controlling nutritional status score. *p*-values < 0.05 are indicated in bold.

### 3.3 Independent association between MIS and objective nutritional indices

Univariable and multivariate linear regression analyses were performed to assess the associations between MIS and objective indices (Table 3). All indices showed significant associations with MIS in univariable analysis. After adjusting for age, sex, dialysis vintage, diabetes, and cardiovascular disease, these associations remained statistically significant. Specifically, ALI (*β* = −0.09, 95% CI: −0.12 to −0.05, *p* < 0.001), PNI (*β* = −0.26, 95% CI: −0.35 to −0.18, *p* < 0.001), and GNRI (*β* = −0.26, 95% CI: −0.33 to −0.20, *p* < 0.001) demonstrated significant inverse associations with MIS. Conversely, CAR (*β* = 0.55, 95% CI: 0.12 to 0.99, *p* = 0.013) and CONUT scores (*β* = 0.69, 95% CI: 0.41 to 0.97, *p* < 0.001) showed significant positive associations with MIS. These results indicated independent correlations between MIS and the objective nutritional indices.

**Table 3:** Univariable and multivariate linear regression analyses of the association between MIS and objective nutritional indices.

Covariates	Univariable analysis			Multivariable analysis		
	*β*	95% CI	*p*	*β*	95% CI	*p*
CAR	0.75	0.35 to 1.15	**<0.001**	0.55	0.12 to 0.99	**0.013**
ALI	−0.09	−0.13 to −0.05	**<0.001**	−0.09	−0.12 to −0.05	**<0.001**
PNI	−0.28	−0.36 to −0.20	**<0.001**	−0.26	−0.35 to −0.18	**<0.001**
GNRI	−0.28	−0.34 to −0.21	**<0.001**	−0.26	−0.33 to −0.20	**<0.001**
CONUT score	0.69	0.41 to 0.97	**<0.001**	0.69	0.41 to 0.97	**<0.001**

CAR, C-reactive protein-to-albumin ratio; ALI, advanced lung cancer inflammation index; PNI, prognostic nutritional index; GNRI, geriatric nutritional risk index; CONUT score, controlling nutritional status score. β, regression coefficient. CI, confidence interval. *p*-values< 0.05 are indicated in bold. Multivariable analysis was adjusted for age, sex, dialysis vintage, diabetes, and cardiovascular disease.

### 3.4 Receiver operating characteristic analysis

**Figure 1:**
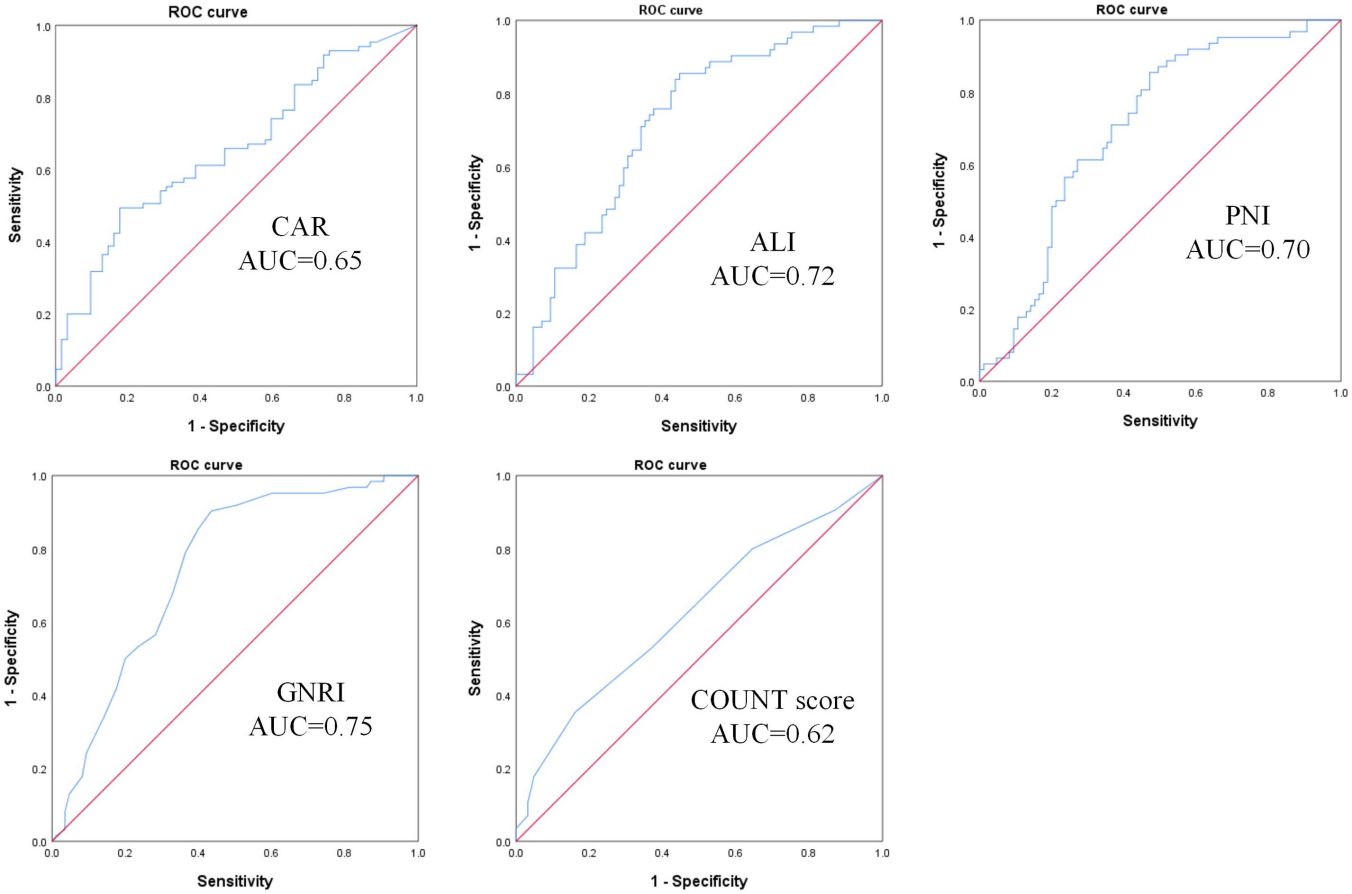
ROC curve for predicting malnutrition-inflammation risk in PD patients using CAR, ALI, PNI, GNRI, and CONUT scores. CAR, C-reactive protein-to-albumin ratio; ALI, advanced lung cancer inflammation index; PNI, prognostic nutritional index; GNRI, geriatric nutritional risk index; CONUT score, controlling nutritional status score.

The predictive performance of five objective indices for malnutrition risk was evaluated by ROC analysis (Table 4, Figure 1). All indices showed significant predictive value. GNRI demonstrated the highest predictive ability with an AUC of 0.75 (95% CI: 0.67–0.83, *p* < 0.001), followed by ALI (AUC = 0.72, 95% CI: 0.64–0.80, *p* < 0.001) and PNI (AUC = 0.70, 95% CI: 0.62–0.79, *p* < 0.001). CAR showed a moderate predictive value (AUC = 0.65, 95% CI: 0.57–0.74, *p* = 0.001). CONUT score had the lowest but still significant predictive value (AUC = 0.62, 95% CI: 0.53–0.71, *p* = 0.01).

**Table 4:** Predictive value of objective indices for malnutrition risk in PD patients.

Characteristic	AUC	*p*	Cutoff value	95% CI	Sensitivity	Specificity
CAR	0.65	0.001	0.14	0.57–0.74	49.40	82.30
ALI	0.72	<0.001	21.63	0.64–0.80	85.50	55.30
PNI	0.70	<0.001	41.96	0.62–0.79	85.50	52.90
GNRI	0.75	<0.001	92.50	0.67–0.83	90.30	56.50
CONUT score	0.62	0.01	4.50	0.53–0.71	35.30	83.90

CAR, C-reactive protein-to-albumin ratio; ALI, advanced lung cancer inflammation index; PNI, prognostic nutritional index; GNRI, geriatric nutritional risk index; CONUT score, controlling nutritional status score.

## 4 Discussion

In this study, we found five objective nutritional indices exhibited significant correlations with MIS and demonstrate substantial predictive utility for malnutrition risk in PD patients. Correlation analyses identified GNRI as the index most strongly correlated with MIS (*r* = −0.558, *p* < 0.001). Furthermore, ROC curve analysis confirmed that GNRI provided the highest predictive accuracy for malnutrition risk (MIS > 5; AUC = 0.75, 95% CI: 0.67–0.83, *p* < 0.001), with an optimal cutoff value of 92.5.

Malnutrition assessed by the CAR, ALI, PNI, GNRI, or CONUT scores has been widely recognized as a significant prognostic indicator in various diseases (11, 12, 18, 19). Studies have demonstrated that elevated CAR levels are significantly associated with increased mortality risk in both PD and HD patients (11, 18). Martins et al. found that elevated CRP was associated with higher MIS in HD patients (9), which is consistent with our findings. We found that CRP and CAR were significantly associated with MIS scores in PD patients. The ROC curve analysis revealed that PD patients with a CAR score of > 0.14 had a significantly higher malnutrition-inflammation risk (MIS > 5).

ALI has been repurposed as a composite marker of malnutrition and systemic inflammation in various chronic diseases with promising predictive value (20–22). A lower ALI value typically indicates malnutrition and chronic inflammatory status and has been identified as an independent risk factor for all-cause and cardiovascular mortality in PD patients (23). Studies in CKD populations have shown that the risk of CKD tended to decrease as ALI increased (24). Zhang et al. have demonstrated that ALI may serve as a prognostic biomarker for CKD patients, especially those with stage III–V disease (12). Our study found a significant inverse association between ALI and MIS in PD patients. Patients with an MIS of ≤5 had significantly higher ALI values than those with an MIS of >5. After adjusting for potential confounders, ALI remained significantly associated with MIS.

PNI, GNRI, and CONUT scores were significantly correlated with MIS in our study. Previous studies have demonstrated that they represent promising prognostic indicators in PD patients (7, 10). The PNI, calculated from serum albumin level and total lymphocyte count, is a valuable predictor of all-cause mortality in HD patients and cardiovascular mortality in PD patients (13, 19). GNRI is calculated based on serum albumin and body weight. CKD patients with lower GNRI have a higher risk of CKD progression, steeper eGFR decline, and higher risk of all-cause mortality (25). Studies have demonstrated that the GNRI is significantly associated with mortality in PD patients and can serve as a straightforward marker for assessing nutritional status in this population (26, 27). The CONUT score, calculated based on serum albumin, total cholesterol, and total peripheral lymphocyte count, has been demonstrated to be a reliable prognostic marker for all-cause mortality, cardiovascular event mortality, and technique failure risk in PD patients (15, 28). Takagi et al. found that the CONUT score might be a simplified surrogate marker of PEW and is comparable to the GNRI in predicting all-cause mortality in patients with CKD who are just initiating dialysis (29).

The five objective indices in our study all include serum albumin as a crucial component, which is a well-known predictor of long-term prognosis in CKD patients (30). Inflammation and malnutrition reduce albumin levels by impairing synthesis and accelerating catabolism (31). Research has shown that hypoalbuminemia is associated with long-term adverse outcomes in ESRD patients, and those with low serum albumin levels face an increased mortality risk (32). However, using serum albumin level alone to assess nutritional status in PD patients is unreliable, since it can be affected by various factors such as inflammation and fluid status (33).

In the general population, obesity is associated with elevated cardiovascular risk and decreased survival. However, a paradoxical ‘obesity paradox’ has been observed in ESRD patients, where higher BMI correlates with improved survival rates (34). This phenomenon may be attributed to the interplay between PEW and chronic inflammation. Specifically, ESRD patients with low BMI are more susceptible to PEW, and malnutrition may exacerbate their vulnerability to inflammatory insults. Consequently, obesity might attenuate the severity of PEW and/or chronic inflammation, potentially conferring survival benefits in this population (34). However, up to 20% of PD patients experience a weight gain of over 10% due to energy in the form of glucose absorbed from PD fluid (35). The research results of Aline et al. showed that higher BMI seems to be associated with higher CRP and impaired immune function in PD patients (35). Therefore, further research is needed to investigate the correlation between BMI and nutritional-inflammation status in PD patients. In this study, we observed a significant negative correlation between BMI and MIS, suggesting that PD patients with lower BMI may face higher risks of MICS.

Body weight or BMI serves as a constituent component of ALI and GNRI. Our study identified the GNRI as the objective index exhibiting the strongest correlation with the MIS in our PD cohort. The GNRI was originally developed by Bouillanne et al. (2005) to assess nutritional risk and predict outcomes in hospitalized elderly patients (36). Although both GNRI and ALI include an anthropometric component, GNRI uniquely integrates the concept of IBW. Specifically, in its calculation, the ratio of current body weight to IBW is capped at 1.0 if a patient’s weight exceeds their IBW. PD patients frequently experience non-nutritional weight increments due to fluid overload (from inadequate ultrafiltration) and systemic glucose absorption from the dialysate (35). By effectively disregarding weight attributable to these confounding factors, the GNRI provides a more accurate and specific estimation of true energy reserves. This feature likely constitutes a key reason for GNRI’s superior performance as a tool for identifying malnutrition-inflammation risk in PD patients. Future studies with larger, multicenter cohorts are warranted to further validate the unique utility of GNRI in the nutritional assessment of this population.

Lymphocyte reduction is associated with inflammation and malnutrition. Studies have shown that lymphocytopenia is an independent predictor of mortality in HD patients (37) and that lower lymphocyte counts are significantly associated with an increased risk of treatment failure in PD-related peritonitis (38). Lymphocyte count serves as a constituent component of ALI, PNI, and CONUT scores. Lower lymphocyte count represents lower ALI, PNI, and a higher CONUT score. NLR is a readily available laboratory marker for predicting systemic inflammation and is significantly elevated in CKD patients with concurrent inflammatory conditions (39). Studies have shown that NLR serves as an independent prognostic factor for all-cause mortality among CKD and dialysis patients (1, 40). Our research indicates that the MIS is significantly positively correlated with NLR and significantly negatively correlated with lymphocyte percentage.

To the best of our knowledge, our study is the first to reveal the association between objective nutritional indices and the subjective assessment tool—MIS—in PD patients. We found that these objective indices may serve as reliable and practical surrogates for nutritional evaluation in PD patients. The primary advantage of indices such as GNRI, ALI, and PNI lies in their derivation from routinely available and inexpensive laboratory parameters—specifically serum albumin, complete blood count (for lymphocyte and neutrophil counts), and basic anthropometric measurements (body weight and height). These tests are standard components of the periodic follow-up for PD patients in most healthcare settings, meaning that these indices can be calculated at negligible additional cost and without requiring extra blood draws. Crucially, these indices are calculated using objective formulas and do not require a specialized assessment by a clinical dietitian or physician. This makes them particularly suitable for resource-limited environments or for large-scale screening programs where comprehensive but time-consuming tools such as the MIS are impractical. Moreover, their application can facilitate the early identification of high-risk patients, prompting timely interventions and thereby potentially improving clinical outcomes in this population.

However, our study also has some limitations. First, it was conducted at a single center with a small sample size, which may affect the robustness and universality of the results. Second, we did not collect data on the use of medications that may influence inflammatory status (e.g., corticosteroids and statins). Consequently, the potential influence of these medications could not be excluded, which might affect the interpretation of our results. Further studies with larger cohorts are needed to identify the most robust objective index for nutritional assessment and establish its optimal thresholds. Prospective studies are also required to determine whether these objective indices can dynamically track changes in nutritional and inflammatory status over time.

## 5 Conclusion

We demonstrated that the objective nutritional indices (CAR, ALI, PNI, GNRI, and CONUT scores) exhibit significant correlations and substantial agreement with the MIS in PD patients. Among them, GNRI shows the strongest association and the highest predictive accuracy for identifying malnutrition-inflammation risk. Compared to the MIS, objective nutritional indices provide benefits such as simplicity, rapid screening, and suitability for large samples. These objective indices hold promise as practical and reliable alternative tools for the rapid assessment of nutritional status in clinical practice, potentially facilitating the early identification of high-risk patients and timely intervention. Future large-scale, multicenter, and prospective studies are needed to validate these findings and to establish standardized application thresholds.

## Data Availability

The raw data supporting the conclusions of this article will be made available by the authors, without undue reservation.

## Glossary

ESKD: End-stage kidney disease
PEW: Protein-energy wasting
MICS: Malnutrition-inflammation complex syndrome
SGA: Subjective Global Assessment
MIS: Malnutrition-Inflammation Score
PD: Peritoneal dialysis
HD: Hemodialysis
CRP: C-reactive protein
CAR: C-reactive protein-to-albumin ratio
ALI: Advanced Lung Cancer Inflammation Index
PNI: Prognostic Nutritional Index
GNRI: Geriatric nutritional risk index
CONUT: Controlling nutritional status
CKD: Chronic kidney disease
BMI: Body mass index
PTH: Parathyroid hormone
TIBC: Total iron binding capacity
LDL-C: Low-density lipoprotein cholesterol
IQR: Interquartile range
NLR: Neutrophil-to-lymphocyte ratio
IBW: Ideal body weight
ROC: Receiver operating characteristic
AUC: Area under the curve
