# Supplementary material for: Association between objective nutritional indices and malnutrition inflammation score in peritoneal dialysis patients
Source: Front Nutr. 2026 Jan 8;12:1713482. doi: 10.3389/fnut.2025.1713482 (PMC12823512; doi:10.3389/fnut.2025.1713482)
Supplement: Supplementary file 1 [file Table_1.DOCX]

**Table S1.** Calculation methods of CONUT score.

| Serum albumin (g/dL) | Albumin score | Total lymphocyte (count/mm³) | TLC score | Total cholesterol (mmol/L) | T-cho score |
| --- | --- | --- | --- | --- | --- |
| ≥ 3.5 | 1 | ≥ 1600 | 0 | ≥ 4.7 | 0 |
| 3.0 - 3.49 | 2 | 1200 - 1599 | 1 | 3.6 - 4.6 | 1 |
| 2.5 - 2.99 | 4 | 800 - 1199 | 2 | 2.6 - 3.5 | 2 |
| < 2.5 | 6 | < 800 | 3 | < 2.6 | 3 |
